# Editorial: Non-linear analysis and machine learning in cardiology

**DOI:** 10.3389/fphys.2023.1183149

**Published:** 2023-04-14

**Authors:** Hans Dierckx, Xiaopeng Zhao, Elena G. Tolkacheva

**Affiliations:** ^1^ Department of Mathematics, KU Leuven Campus Kortrijk (KULAK), Kortrijk, Belgium; ^2^ iSi Health - KU Leuven Institute of Physics-based Modeling for in silico Health, Leuven, Belgium; ^3^ Department of Mechanical, Aerospace and Biomedical Engineering, University of Tennessee, Knoxville, TN, United States; ^4^ Biomedical Engineering Department, University of Minnesota, Minneapolis, MN, United States; ^5^ Institute for Engineering in Medicine, University of Minnesota, Minneapolis, MN, United States; ^6^ Lillehei Heart Institute, University of Minnesota, Minneapolis, MN, United States

**Keywords:** cardiology (basic/technical), machine learning, ML, data analysis, non-linear analysis methodologies, multiscale modeling and analysis

Cardiovascular diseases remain a major cause of death accounting for about 30% of death worldwide according to the World Health Organization. Over the past decades, various interdisciplinary approaches have been developed *via* close collaboration between clinicians, engineers and basic scientists to reveal fundamental mechanisms and develop approaches to perform analysis of cardiac abnormalities. The analysis can be performed on different available measurements in patients, which can be electrical (*via* electrode recordings), mechanical (*via* imaging), hemodynamic, or even entail overall patient data. As the heart is a complex dynamical system, the simple linear approaches are not always successful, and advances in non-linear signal processing and machine learning (ML) guided analysis techniques could lead to a better understanding, diagnosis and treatment of cardiac diseases.

In addition to traditional methods of ECG analysis based on filtering, spectral analysis and statistical approaches, various non-linear dynamic modeling and ML approaches have been recently developed to perform quantitative analysis of electrical signals from the heart. The contributions to this Research Topic are covering recent advances and novel research trends in such approaches, aiming to discriminate between normal and abnormal cardiac rhythms, to offer insights into fundamental processes, or even predict the future evolution of a cardiac cell from time series.

The fundamental biophysical level is key to understand the collective behaviour of myocytes during arrhythmias. (Béland et al.) improved the analysis of videomicroscopy of cultured myocytes, by assessing not only the rate of activity but also the contractile properties of the beating cells. Such approach is motivated by the use of cell cultures to high-throughput screening of cardiotoxicity in drug development. (Nowak et al.) performed numerical simulations to investigate the effect of ephaptic coupling on conduction velocity in the tissue, and reported a non-linear, biphasic dependency on cell size. They concluded that developmental changes predict changes in cardiac conduction. Interesting results of multiscale modeling were presented by (Shahi et al.), who demonstrated that ML offers a way to construct predictive models for local electrical activity, when cardiac action potentials are predicted either using recurrent neural networks or reservoir computing.

At the tissue level, an important characteristic in cardiac excitation patterns is the activation time. (Ramlugun et al.) presented a comprehensive framework to extract the activation time from optical mapping signals, accounting for the repetitive activation during complex arrhythmia. An alternative representation of activation times is phase mapping, which is a well-established technique to determine the location of phase singularities during recordings of chaotic regimes such as ventricular fibrillation. However, in a clinical context, the phase maps are often noisy or sparse. To enable phase singularity identification in these cases, (Lebert et al.) proposed a self-supervised deep-learning approach that immediately recovers the phase maps from short spatio-temporal sequences of electrical data. Meanwhile, (Arno et al.) assessed shortcomings of traditional phase analysis, integrated the concepts of linear rotor cores and conduction block lines into a structure called “phase defect lines”, and showed their presence in optical mapping recordings.

Moving one step further towards clinical applications, two contributions focused on image analysis. (Wu et al.) adopted a deep neural network approach to find objects of interest in medical images without the need for predefined thresholds, and applied it to cardiac images in movie data. (Yang et al.) described a novel way to extract the characteristics of vortex flows in the right atrium, from phase-contrast MRI images.

Non-linear analysis can also be used to support or guide specific therapy. For instance, (Ravikumar et al.) developed a similarity score from analysis of clinical intracardiac electrograms to identify active sites of atrial arrhythmias for potential guiding for ablation therapy. (Khamzin et al.) presented a predictive model for the outcome of cardiac resynchronization therapy. They found that including computational modeling results increased the quality of predictions compared to a pure ML approach from clinical data only.

Finally, two papers in this Research Topic described advances in improving the analysis of ECG signals. (Zheng et al.) developed a ML approach to recover the location of arrhythmia triggers based on the 12-lead ECG. Their method can help to plan ablation procedures for triggers near the right or left-ventricular outflow tracts. (Shinoda et al.) utilized Poincaré mapping and recurrence quantification analysis to characterize stochasticity vs. chaotic dynamics in the heart, to distinguish between naturally varying ECG characteristics and a pathological condition. Their method is then applied to a Parkinson’s disease animal model.

Non-linear signal processing and ML guided analysis techniques have played a key role in better understanding, diagnosing, and treating cardiovascular diseases. The contributions to this Research Topic cover recent advances and novel research trends in such approaches, with a focus on discriminating between normal and abnormal cardiac rhythms, offering insights into fundamental processes, or even predicting the future evolution of a cardiac cell from time series. These advances span from analyzing individual myocytes to improving the analysis of ECG signals, to extracting the characteristics of vortex flows in the right atrium from phase-contrast MRI images.

The studies presented in this Research Topic demonstrate the utility of ML approaches in clinical applications for decision support and improving patient outcomes. In the future, the proposed frameworks need to be adjusted to address various disease monitoring and subsequent disease prediction. Further development of various signal processing and data analysis techniques based on different non-linear and ML approaches are also warranted for multiple wearable sensors.

